# Impact of Standardized Discharge Guidelines on Outpatient Follow-Up for Pediatric Asthma Patients: A Retrospective Study

**DOI:** 10.7759/cureus.112900

**Published:** 2026-07-18

**Authors:** Philip Roth, Madison McDaniel, Nyra Ahmed, Ratheya Rajakumar, Shireen Agrawal, Roshini Murugadass, Emma Hillis, Michelle Puzdrakiewicz

**Affiliations:** 1 Ochsner Clinical School, The University of Queensland, New Orleans, USA; 2 Biostatistics and Epidemiology, Ochsner Medical Center, New Orleans, USA; 3 Pediatrics, Ochsner Medical Center, New Orleans, USA

**Keywords:** asthma, consensus, patient discharge, quality improvement, respiration disorders

## Abstract

Introduction

Asthma is a chronic respiratory condition and a leading cause of pediatric hospitalization. Asthma education and proper follow-up after discharge improve asthma control and reduce exacerbations. This study evaluates whether standardized discharge guidelines improved outpatient follow-up.

Methods

A retrospective chart review was conducted on pediatric patients hospitalized with asthma between March 10 and September 9, 2024, for the pre-intervention period and between September 10, 2024, and March 10, 2025, for the post-intervention period. Outcomes from the six months before implementation of the new asthma discharge guidelines on September 10, 2024 (n = 86), were compared with those from the six months after implementation (n = 77). Primary outcomes were defined as whether referrals and scheduled follow-ups with primary care physicians (PCPs) and/or specialists were provided at discharge.

Results

Implementation of the consensus was not associated with significant changes in PCP follow-up timing or scheduling. The median time to the PCP appointment did not significantly differ between groups and remained three days before and after the intervention (p = 0.4959). Rates of scheduled PCP follow-up at discharge were not significantly different (52.33% vs. 42.86%; p = 0.2270). In contrast, scheduled specialist follow-up at discharge significantly improved (12.79% vs. 46.75%; p < 0.0001), as did the scheduling of specialist appointments within 30 days of discharge (13.95% vs. 33.77%; p = 0.0001).

Conclusion

The standardized discharge protocol correlated with more timely, scheduled follow-up with specialists. Future studies should evaluate whether the increase in specialist appointments reduces future asthma exacerbations and hospital admissions.

## Introduction

Asthma is a chronic inflammatory respiratory condition that affects millions of patients around the globe. Symptoms of asthma include wheezing, shortness of breath, and cough, which can be triggered by various stimuli and range in severity [1]. According to the Global Initiative for Asthma (GINA), pediatric patients at high risk for asthma exacerbations are those with prior intensive care admission for asthma exacerbations, one or more severe exacerbations within one year, overuse of short-acting beta agonists, and an FEV1 less than 60% of the predicted value [2]. Asthma management is based on long-term control of symptoms, which involves assessment of symptoms and risk factors, review of both pharmacological and nonpharmacological treatment, and response to treatment [1,2].

Effective management and discharge planning have been shown to improve patients' asthma and reduce the rates of exacerbation [2]. One study has shown that effective therapeutic management and patient education significantly reduced asthma attacks and lowered emergency room visits [3]. Another study found that the implementation of allergy clinic-based interventions, including proactive scheduling, increased post-hospitalization follow-up rates and decreased asthma readmission rates [4].

Several institutions in the United States have developed asthma discharge protocols that use evidence-based guidelines. Children's Hospital Colorado, Children's Healthcare of Atlanta, Seattle Children's Hospital, and the University of California, San Francisco, developed asthma discharge protocols through local multidisciplinary consensus among hospital clinicians and stakeholders, while Children's Hospital of Philadelphia was guided by the Focused Updates to the Asthma Management Guidelines, and Children's Minnesota developed its guidelines based on GINA and several other institutional guidelines [5-10].

The Ochsner hospital system consists of 46 hospitals whose population includes both metropolitan and rural patients from Louisiana as well as Mississippi. In September 2024, the Ochsner pediatric hospitalist group developed a Delphi consensus, a structured process in which experts iteratively review and refine recommendations to achieve group agreement, on asthma discharge management to standardize care for pediatric patients admitted for asthma exacerbations (Appendix 1). The discharge management guidelines emphasized follow-up with a primary care physician (PCP) within two to three days of discharge and referral to a pulmonology or allergy-immunology specialist within one month of discharge for high-risk patients.

This study aims to assess whether implementing the new discharge management guidelines has improved follow-up care. The primary outcome was to determine whether patients were provided with a scheduled outpatient follow-up with their PCP within two to three days and, if the patient was considered at high risk, whether a referral to a pulmonologist, allergist, or immunologist was scheduled within one month after discharge, in accordance with the Delphi consensus.

## Materials and methods

Study design 

This study was a retrospective chart review designed as a quality improvement project conducted at Ochsner Medical Center and focused on pediatric patients admitted for asthma exacerbation. The Plan, Do, Study, Act (PDSA) framework was used for this study. The Plan and Do phases (development and implementation of the Ochsner Delphi Consensus: Asthma Implementation) had already been completed, and this study focused on the Study and Act phases. Data were analyzed from patients seen six months prior to and six months after the implementation of the new discharge management guidelines on September 10, 2024, corresponding to the study period from March 10, 2024, to March 10, 2025. This study posed minimal risk to participants, and, following IRB approval, pediatric patients were identified using the Slicer Dicer tool in Epic, and eligible charts were selected.

Appointments for both PCP and specialist follow-ups were confirmed to determine if they were scheduled during discharge planning, and patient attendance was verified through follow-up documentation. The rates of these referrals and scheduled appointments during the six months before and after implementation of the new discharge management guidelines were compared. For specialist follow-up, only patients who were designated as high-risk asthmatics, had significant comorbidities (allergies, eczema, obesity, obstructive sleep apnea, or daily smoke exposure), or required multiple courses of steroids within a one-year period received follow-up appointments with specialists. High-risk asthmatics were classified as patients with any history of PICU admission for asthma exacerbation, more than two hospital admissions for asthma within one year, poorly controlled asthma according to National Heart, Lung, and Blood Institute guidelines (daytime symptoms more than two days a week, rescue inhaler use for symptoms more than two days a week, waking at night with symptoms three or more times a month, or any limitation of normal daily activities), or a Pediatric Asthma Severity Score (PASS) greater than or equal to 12 after one treatment. The patients' charts were kept confidential and were recorded and managed in Microsoft Excel (Microsoft® Corp., Redmond, WA, USA), using password protection for additional security. 

Study participants 

Patients were included if they were between 2 and 18 years of age, admitted to Ochsner Main Campus Hospital, and received treatment for asthma during hospitalization as either a primary or secondary diagnosis. All charts retrieved from Slicer Dicer that did not meet these criteria were excluded. A total of 163 patients were included, with 86 patients admitted before and 77 patients admitted after the intervention.

Statistical analysis

Categorical variables were compared using chi-square tests or Fisher's exact tests, as appropriate. Continuous outcomes were compared using Wilcoxon rank-sum tests. Patients with missing data for a given outcome were excluded from the corresponding analysis. All statistical tests were evaluated at the 0.05 significance level, and all analyses were conducted using SAS version 9.4 (SAS Institute Inc., Cary, NC, USA).

## Results

The total number of patients included in the analysis was 163, with 77.9% of the patients admitted with a primary asthma diagnosis, while the remaining 22.1% were admitted with asthma as a secondary diagnosis. The distribution of asthma severity was classified as intermittent, mild, moderate, and severe (Table 1).

**Table 1:** Patient charateristics before and after intervention ^a^ Categorical variables were compared using Pearson’s chi-square test or Fisher’s exact test, as appropriate, and continuous variables were compared using the Wilcoxon rank-sum test. Pearson X² statistics and Wilcoxon standardized Z statistics are reported. For Fisher’s exact test, only the exact p-value is reported.

		All N = 163	Before Intervention N = 86	After Intervention N = 77	Test Statistic^a^	p-value
							Diagnosis	Primary Asthma Diagnosis, n(%)	127 (77.91)	62 (72.09)	65 (84.42)	X^2^ = 3.58	0.0583
Secondary Asthma Diagnosis, n(%)	36 (22.09)	24 (27.91)	12 (15.58)										
Severity	Intermittent, n(%)	27 (16.56)	19 (22.09)	8 (10.39)	X^2^ = 4.17	0.2433
	Mild, n(%)	36 (22.09)	18 (20.93)	18 (23.38)		
	Moderate, n(%)	41 (25.15)	21 (24.42)	20 (25.97)		
	Severe, n(%)	59 (36.20)	28 (32.56)	31 (40.26)		
Age	Median (IQR)	7.0 (4.0, 10.0)	6.0 (4.0, 11.0)	7.0 (4.0, 10.0)	Z = -0.24	0.8075
Gender	Female, n(%)	81 (49.69)	53 (61.63)	28 (36.36)	X^2^ = 10.37	0.0013
	Male, n(%)	82 (50.31)	33 (38.37)	49 (63.64)		

For PCP follow-up after discharge, the median time was unchanged after the intervention at 3.0 days (p = 0.4959; Table 2). Sixty-seven patients were excluded because no PCP follow-up appointment was scheduled. In contrast, follow-up with a pulmonologist or allergist occurred significantly sooner after implementation (median, 31 days vs. 14 days; p = 0.0169; Table 2). PCP follow-up scheduled at discharge (52.33% vs. 42.86%, p = 0.2270; Table 2) and attendance at PCP visits (31.40% vs. 33.77%, p = 0.2446; Table 2) showed no significant difference.

**Table 2:** Follow-up care outcomes before and after intervention ^a^ Categorical variables were compared using Pearson’s chi-square test or Fisher’s exact test, as appropriate, and continuous variables using the Wilcoxon rank-sum test. Pearson X^2^ statistics and Wilcoxon standardized Z statistics are reported. For Fisher’s exact test, only the exact p-value is reported. ^b^ Patients were not included if they were not scheduled for a PCP follow-up appointment. ^c^ Patients who were classified as high-risk asthmatics, have multiple comorbidities, or require multiple steroid courses within one year received a referral to pulmonology or allergy immunology. ^d^ Patients who scheduled an appointment with pulmonology or allergy immunology were included in the category. All who did not receive a specialist appointment were excluded. PCP: primary care physician

		All N = 163	Before Intervention N = 86	After Intervention N = 77	Test Statistic^a^	p-value
							PCP Follow-Up Scheduled at Discharge	No, n(%)	85 (52.15)	41 (47.67)	44 (57.14)	X^2^ = 1.46	0.2270
Yes, n(%)	78 (47.85)	45 (52.33)	33 (42.86)										
Scheduled PCP Follow-Up Within 2-3 Days	No, n(%)	109 (66.87)	57 (66.28)	52 (67.53)	X^2^ = 0.03	0.8652
	Yes, n(%)	54 (33.13)	29 (33.72)	25 (32.47)		
Days after discharge follow-up appointment with the PCP was scheduled	Median (IQR)	3.0 (2.0, 6.0)	3.0 (2.0, 6.0)	3.0 (2.0, 6.0)	Z = -0.68	0.4959
	Excluded ^b^, n(%)	67 (41.10)	32 (37.21)	35 (45.45)		
Attended follow-up (PCP)	No, n(%)	43 (26.38)	27 (31.40)	16 (20.78)	X^2^ = 1.35	0.2446
	Yes, n(%)	53 (32.52)	27 (31.40)	26 (33.77)		
	Excluded ^b^, n(%)	67 (41.10)	32 (37.21)	35 (45.00)		
Referral with pulmonologist or allergy-immunology scheduled at discharge	No, n(%)	44 (26.99)	39 (45.35)	5 (6.49)	X^2^ = 39.06	<0.0001
	Yes, n(%)	47 (28.83)	11 (12.79)	36 (46.75)		
	Excluded ^c^, n(%)	72 (44.17)	36 (41.86)	36 (46.75)		
Scheduled pulmonologist and/or allergy-immunology follow-up within 30 days	No, n(%)	53 (32.52)	38 (44.19)	15 (19.48)	X^2^ = 14.39	0.0001
	Yes, n(%)	38 (23.31)	12 (13.95)	26 (33.77)		
	Excluded ^c^, n(%)	72 (44.17)	36 (41.86)	36 (46.75)		
Days after the discharge, a follow-up appointment with pulmonology and/or allergy/immunology was scheduled	Median (IQR)	21.0 (9.0, 42.0)	31.0 (10.0, 53.0)	14.0 (6.5, 24.0)	Z = -2.39	0.0169
	Excluded ^d^, n(%)	94 (57.67)	49 (56.98)	45 (58.44)		
Attended follow-up (pulmonology/allergy-immunology)	No, n(%)	18 (11.04)	12 (13.95)	6 (7.79)	X^2^ = 1.67	0.1968
	Yes, n(%)	51 (31.29)	25 (29.07)	26 (33.77)		
	Excluded ^d^, n(%)	94 (57.67)	49 (56.98)	45 (58.44)		
Referral reason	ICU, n(%)	58 (35.58)	27 (31.40)	31 (40.26)	-	0.1034
	Comorbidities, n(%)	26 (15.95)	18 (20.93)	8 (10.39)		
	Admissions, n(%)	7 (4.29)	5 (5.81)	2 (2.60)		
	Excluded ^c^, n(%)	72 (44.17)	36 (41.86)	36 (46.75)		

Seventy-two patients were excluded from the specialist follow-up analysis because they did not meet the criteria for high-risk asthma requiring specialist referral. Referrals to pulmonologists or allergy-immunologists at discharge were significantly more common after implementation of the standardized discharge protocol (12.79% vs. 46.75%, p < 0.0001; Table 2). Scheduled pulmonology or allergy-immunology follow-up within 30 days also increased significantly (13.95% vs. 33.77%, p = 0.0001; Table 2). The number of patients who attended their follow-up appointments with pulmonology or allergy-immunology was not statistically significant (p = 0.1968; Table 2).

## Discussion

This study found that structured intervention for pediatric asthma patients resulted in significant improvements in follow-up with pulmonologists or allergy-immunologists, but it had no impact on PCP follow-ups. PCP follow-up scheduled at discharge and PCP follow-up within two to three days were not statistically significant. The median number of days after discharge that a PCP follow-up appointment was scheduled was unchanged following the implementation of the standardized discharge protocol, at 3.0 days (p = 0.4959). The absence of significant improvement in follow-up appointment scheduling for PCP referrals may result from unrealistic expectations from pediatric hospitalists due to limited clinic availability within a short timeframe of just two to three days.

There were several patients who were not followed by a primary pediatrician within the Ochsner Health System and therefore could not receive a referral through the EMR. These patients may have received a recommendation to follow up in their discharge instructions and could have followed up with their provider. However, no documentation of a follow-up visit could be found, which constitutes an unmodifiable factor that the discharge management guidelines could not address. Some patients may have had a PCP appointment scheduled at discharge within two to three days, which was later rescheduled to a later follow-up date. Because documentation of these changes was unavailable at the time of chart review, these patients may not have been recorded as having follow-up appointments scheduled at discharge within the two-to-three-day timeframe and could therefore represent false negatives, as only the rescheduled date would have been recorded. 

A previous study at the University of Colorado Hospital showed that a lack of timely follow-up with a PCP was associated with higher hospital readmission rates for the same condition [11]. PCPs play an essential role in preventing and managing asthma exacerbations, and most patients can be managed by primary care worldwide [2,11,12]. PCPs can prevent and manage asthma exacerbations by encouraging patient adherence, teaching and assessing correct inhaler techniques, and emphasizing the importance of reliever use and inhaled corticosteroids [13]. There is variability among the protocols of the reviewed institutions regarding the recommended timeframe for outpatient follow-up with the patient’s PCP. Children’s Minnesota and Children’s Hospital of Atlanta recommend that follow-up within two to three days of discharge is ideal, whereas Children’s Hospital of Colorado recommends outpatient follow-up within 10 days [6-8].

Referral to pulmonology and allergy-immunology at discharge improved after the implementation of the standardized discharge protocol (p < 0.0001), as did the time from discharge to the scheduled appointment (p = 0.0001). Patients were scheduled for specialist follow-up more often and sooner after discharge. Given that the pulmonologists and allergists were within the same health system, this may have led to better coordination and easier scheduling, whereas follow-up appointments with PCPs outside the Ochsner Health System may have been more challenging to schedule and would have relied on the patient’s guardian to arrange the appointment. 

Studies have shown that the implementation of various reminder systems and incentives has improved follow-up attendance after hospitalization. Ruffner et al. found that the use of telephone reminders increased follow-up to an allergist for asthma exacerbation [4]. Similarly, Baren et al. used telephone reminders and transportation assistance, which significantly increased the likelihood of follow-up [14,15]. A study by Smith et al. provided telephone coaching and monetary incentives to encourage PCP follow-up after emergency room visits for asthma [16]. The addition of built-in reminders leading up to follow-up appointments that were scheduled at discharge may improve attendance rates. Follow-up with pulmonology or allergy clinics was also recommended by the other institutions' protocols. Several pediatric institutions have implemented asthma discharge protocols emphasizing risk-stratified follow-up with primary care, pulmonology, and allergy specialists, along with standardized patient education and post-discharge care coordination [5-10].

There are limitations to this study. The standardized discharge guidelines for pediatric asthma exacerbations were only rolled out at the Ochsner Main Campus hospital. Patient selection was limited to those treated at Ochsner Main Campus, which may have limited the sample size and decreased the power of this study. The short duration of six months before and after the implementation of the new discharge guidelines likely contributed to this. The two groups were evaluated during two different seasons, which may have contributed to the significant differences in the severity of asthma exacerbations and gender distribution between the groups before and after the implementation of the standardized asthma discharge protocol. This study did not assess the long-term clinical outcomes regarding follow-up with specialists after discharge to determine whether there was any improvement in preventing future asthma exacerbations.

## Conclusions

In conclusion, the results of this study suggest improvement in specialist follow-up after discharge; however, no significant improvement in PCP follow-up was observed. This further suggests that the implementation of the new discharge management guidelines for asthma exacerbations was partially successful in accomplishing its recommendations for a standardized asthma discharge protocol for pediatric patients, based on results showing timelier scheduled follow-up with specialists. Future recommendations include reviewing and implementing a new Delphi consensus, with an additional focus on asthma education at discharge and evaluating whether the increase in specialist follow-up decreases future asthma exacerbation admissions.

## Appendices

Appendix 1

Delphi Consensus: Asthma, Final Report

Ochsner pediatric hospitalist departmental guidelines for discharge management regarding pediatric patients admitted with asthma exacerbation.

Asthmatic Patient Discharge Plan

· PCP follow-up scheduled within 2-3 days of discharge with allergy/pulmonology follow-up within one month if the patient is considered a high-risk asthmatic.

· Patients should receive a completed, written asthma action plan prior to discharge.

· The asthma action plan should be included in the discharge summary for review by the PCP.

· Patients and families should receive asthma education and medication training by a designated asthma educator prior to discharge.

High-risk asthmatics are defined as:

· Any history of PICU admission for asthma exacerbation

· Greater than two hospital admissions within a year for asthma exacerbation

· Patient with poorly controlled asthma per NHLBI guidelines*

· Patient with severe asthma exacerbation: determined by a PASS score greater than or equal to 12 after one treatment.

Consider pulmonary or allergy follow-up if the patient has:

· Required multiple steroid courses within a year

· Has significant asthma comorbidities (allergies, eczema, obesity, OSA, daily smoke exposure, etc.)
